# Camera Traps Uncover the Behavioral Ecology of an Endemic, Cryptic Monkey Species in the Congo Basin

**DOI:** 10.3390/ani13111819

**Published:** 2023-05-31

**Authors:** Charlene S. Fournier, Steven McPhee, Junior D. Amboko, Kate M. Detwiler

**Affiliations:** 1 Department of Biological Sciences, Florida Atlantic University, Boca Raton, FL 33431, USA; 2 Department of Anthropology, Florida Atlantic University, Boca Raton, FL 33431, USA

**Keywords:** *Cercopithecus*, guenon, birth seasonality, terrestrial locomotion, group size

## Abstract

**Simple Summary:**

Over the past decade, camera traps have proven to be a valuable and efficient tool to collect data on cryptic and endangered animal species. Since its description in 2012, terrestrial camera trap surveys have been conducted on *Cercopithecus lomamiensis* (common name lesula), an endemic primate species of the central Congo Basin in the Democratic Republic of the Congo. The objective of this study was to use camera trap data to expand knowledge on the behavioral ecology of this cryptic species. We established two systematic camera trap grids inside the Lomami National Park and one outside the park in the buffer zone, where animals are heavily hunted. We confirmed that lesula’s locomotor behavior is highly terrestrial, has a diurnal activity pattern, and lives in medium–large groups with one adult male, multiple adult females and their juveniles. Observations of young infants suggest peaks in births during the wet season. Our study provides new information about the behavioral ecology of this little-studied primate species, generating species-specific knowledge of a threatened species for successful conservation planning.

**Abstract:**

Guenons are the most diverse clade of African primates, and many species living within the core of the Congo Basin rainforest are still understudied. The recently described guenon species, *Cercopithecus lomamiensis*, known as lesula, is a cryptic, semi-terrestrial species endemic to the central Congo Basin in the Democratic Republic of the Congo. The recent IUCN Red List Assessment recognizes lesula’s risk of extinction in the wild as Vulnerable. The objective of our study was to use camera traps to expand knowledge on the behavioral ecology of lesula. We conducted three systematic, terrestrial camera trap (CT) surveys within Lomami National Park and buffer zone (Okulu: 2013; Losekola: 2014; E15: 2015). We accumulated 598 independent events of lesula over 5960 CT days from 92 CTs. Typical of *Cercopithecus* species, camera trap videos reveal that lesula has a diurnal activity pattern, birth seasonality, a group size of up to 32 individuals, and social organization with female philopatry and male dispersal. Results also suggest that lesula are highly terrestrial, distinguishing them from other *Cercopithecus* species, which are mostly arboreal. Our study provides new information about the behavioral ecology of this little-studied primate, generating species-specific knowledge of a threatened species for successful conservation planning.

## 1. Introduction

The lesula monkey (*Cercopithecus lomamiensis*) was described in 2012 as a semi-terrestrial rainforest guenon species endemic to the Tshuapa, Lomami, and Lualaba (TL2) Rivers Landscape in the central Congo Basin [1]. Lesula is a member of the Cercopithecini tribe, which is the most diverse and understudied radiation of Old-World monkeys [2,3]. The TL2 Landscape supports high guenon diversity with six distinct species: *Cercopithecus wolfi*, *C. ascanius*, *C. mitis*, *C. neglectus*, *C. lomamiensis*, and *Chlorocebus dryas* [1,4]. The scientific discovery of lesula increased our knowledge of the diversity and endemism of the Congo Basin’s primates. Due to bushmeat hunting in its range, lesula is listed as Vulnerable in the IUCN Red List of Threatened Species [5].

**Figure 1:**
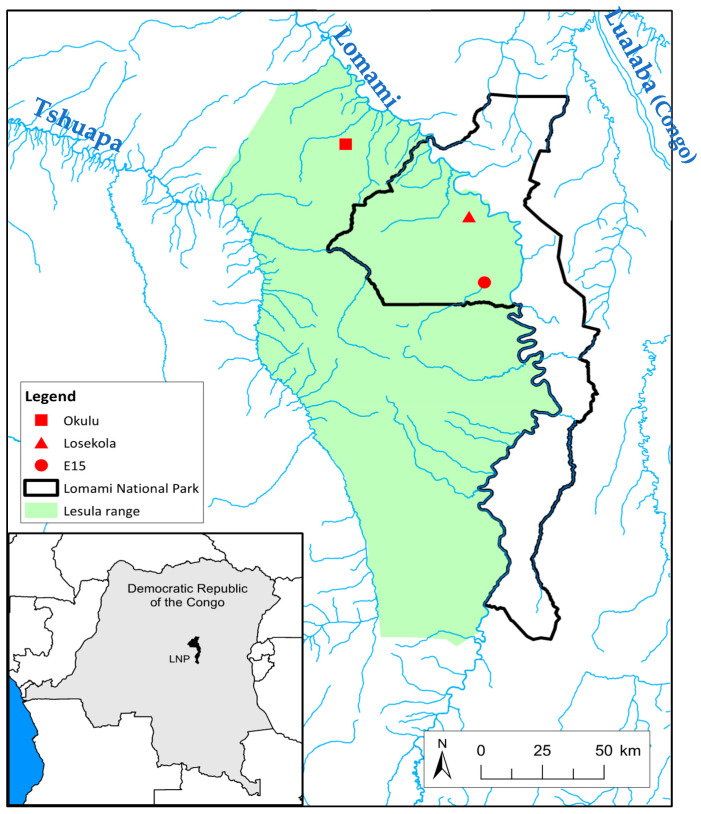
Map inset: Lomami National Park (LNP) in the Democratic Republic of the Congo. Enlarged map: The three survey sites in LNP and buffer zone within the lesula (*C. lomamiensis*) range with the three major rivers bordering the area (Tshuapa, Lomami, and Lualaba Rivers).

Genetic analyses determined that lesula is a sister species to *Cercopithecus hamlyni* [1]. *C. hamlyni* lives on the east bank of the Lualaba (Congo) River, whereas *C. lomamiensis* lives on the west bank of the Lomami River (Figure 1) [1]. Neither species has been confirmed in the Lomami–Lualaba interfluve. *C. lomamiensis* and *C. hamlyni* have recently diverged and have a geographic range that does not overlap, suggesting that an allopatric speciation event occurred in the past, separating the ancestral population [6]. However, we have little understanding of the ecological and behavioral adaptations of *C. hamlyni* and *C. lomamiensis*—the only two species of the *hamlyni* species-group. To date, few field studies have been conducted on this lineage and minimal information is available in the literature [1,7,8,9,10,11]. Furthermore, the cryptic behavior of these species creates additional challenges when applying traditional observational methods in the field. To address a lack of comprehensive ecological knowledge on this lineage, we designed an intensive camera trap study focusing on lesula to complement initial field observations of its behavioral ecology.

Members of the *Cercopithecus* genus are characterized as arboreal guenons; however, the *hamlyni* species-group represents a unique phylogenetic lineage with an ecological niche that is thought to be mostly terrestrial [1,12]. The incongruent pattern of lesula and *C. hamlyni’s* terrestriality within a clade of arboreal monkeys raises questions about the evolution of terrestriality in guenons and the distinctiveness of the ecology and behavior of this species-group [12]. No behavioral study has been done to evaluate the degree of terrestriality of the lesula monkey; thus, we designed a camera trap study to determine lesula’s main mode of locomotion. We predicted that lesula would be captured on the forest floor at a higher rate than other sympatric arboreal species.

As an Old-World monkey species, lesula should be strictly diurnal; however, the morphological analysis of lesula skulls shows a unique shape with orbits larger than other guenon species [1]. As morphology is linked to function, a study looking at 60 extant primates found that nocturnal species exhibit proportionally larger orbits than diurnal species [13]. In addition, patrol teams in Lomami National Park (LNP) frequently report the lesula’s pre-dawn boom calls on listening point surveys, which could suggest activity during crepuscular hours [1,14]. Other studies have used camera traps and satellite telemetry to report activity beyond daylight hours, where fluctuations in food resources, overnight temperature, thermoregulation, or predation threats play a role in activity patterns of mammals [15,16,17,18]. In this study, we assessed lesula captures over a 24 h period to determine if a divergent activity pattern exists in this species (crepuscular or cathemeral).

Guenon species are also known to exhibit reproductive and birth seasonality [19,20]. However, female primates with newborn infants are known to be highly protective and cryptic [21,22], and no field observations of lesula infants have been reported. Thus, we used a systematic approach to investigate a possible birth seasonality in the species, which may be related to months of high precipitation. Studies have found that rainfall and food availability are likely to determine the timing of reproduction when no marked annual variations in daylength or temperature are observed [19,23]. In environments that experience two wet seasons per year, guenon birth seasons often last between four to six months and occur within the first half of the long, wet season [19].

Finally, guenons usually live in medium-sized social groups, from 10 to 40 individuals, composed of a single male and multiple females with their juveniles [20,24]. Females are philopatric and males disperse when they reach sexual maturity [25]. As a *Cercopithecus* species, lesula should follow the same social organization and dispersal pattern; however, there are few data available on lesula group size and composition. Field observations from the initial scientific study on the species provided a maximum number of five individuals per group and no information on group composition [1]. Lesula, living in small family groups, may be an exception among the more social *Cercopithecus* species, similar to De Brazza’s monkeys (*C. neglectus*) [26]. However, a small group size may also be the result of observer bias due to limited surveys in a challenging environment. The species’ cryptic behavior makes it difficult to conduct group counts in their forest habitat. Thus, we used camera traps as the best approach to estimate group counts from non-habituated lesula individuals without any disturbance to their social groups.

The first scientific report of lesula in 2012 increased the diversity of the guenon radiation and highlighted the need for species-specific research on lesula and other guenon taxa to better understand the complex behavioral and ecological traits found within the tribe. There is a growing recognition of the importance of incorporating life history and behavioral data of threatened species into wildlife management practices [27]. There is often a disconnect between scientific knowledge of threatened, endemic species and management decisions made on their behalf. On the ground, conservationists benefit when they can incorporate scientific data into decision making about protection activities and allocation of resources [28]. For example, life history data could inform wildlife managers when to increase patrol intensity, such as during birth or breeding seasons. In addition, small terrestrial mammals are at risk of snares, so understanding their movement ecology can improve wildlife protection strategies [14,29].

Camera traps are non-invasive and able to collect ecological and behavioral data on rare or non-habituated populations without disturbing individual behaviors or the dynamics of a group. In this study, we used camera trapping methods, which have proven to be cost- and time-effective in numerous mammal studies, to collect behavioral data [30,31,32,33]. The objectives of our study were to (1) examine the substrate use of lesula through terrestrial camera traps, (2) confirm lesula diurnality and rule out any possible divergent activity pattern, (3) investigate a possible birth seasonality occurring during the wet season, and (4) document complete group counts to determine the minimum group size and composition. The overall goal of the study was to increase species-specific knowledge of lesula, providing new scientific data for use in conservation planning. To this end, preliminary results from this study were incorporated into the recent IUCN Red List assessment of lesula [5].

## 2. Materials and Methods

### 2.1. Ethics Statement

The Institut Congolais pour la Conservation de la Nature (ICCN) granted permission to conduct research within LNP and provided park guards and interns to accompany the field team. We surveyed a forest in the buffer zone with authorization from the sector chief of the Yawende Lolo Sector, Tshopo Province.

### 2.2. Camera Trap Surveys

#### 2.2.1. Study Area

The TL2 Landscape, a 40,000 km^2^ conservation area in the Democratic Republic of the Congo, is comprised of the Lomami National Park and its buffer zone [1]. The Lomami National Park (2°0′0″ S, 25°2′0″ E), an 8874 km^2^ area of contiguous tropical lowland forest and seasonally flooded prairies, was gazetted on 19 July 2016 to protect threatened species from intensive hunting (Figure 1) [34]. Bushmeat hunting is legal in the buffer zone with the implementation of hunting seasons and regulations. The area has a tropical climate with two wet seasons per year. Due to its location near the equator, the day/night duration is close to 12:12 h year-round. The TL2 region is a critical conservation landscape for animal diversity, such as bonobos (*Pan paniscus*), Congo peafowls (*Afropavo congensis*), forest elephants (*Loxodonta cyclotis*), okapi (*Okapi johnstoni*), and pangolins (*Smutsia gigantea*, *Phataginus tricuspis* and *P. tetradactyla*) [1].

#### 2.2.2. Survey Sites

We established three research areas where the target species *Cercopithecus lomamiensis* had been reported. Two of the survey sites (Losekola: −1°23′27.564″ S, 24°58′27.732″ E and E15: −1°38′30.516″ S, 25°1′58.26″ E) were within the newly formed LNP. The third survey site was in the buffer zone (Okulu: −1°6′22.608″ S, 24°31′25.572″ E), where animals are intensively hunted (Figure 1). The Okulu survey, named after the closest river tributary, was chosen due to its mixed forest, topographical features, and distance from local villages (<10 km) [35]. The LNP is divided into management areas that are regularly patrolled by park guards. Two management areas, Losekola and E15, are sites where patrol teams frequently report lesula’s dawn boom calls.

#### 2.2.3. Survey Design

We conducted three terrestrial, systematic camera trap surveys over a three-year period. We generated systematic survey grids in ArcGIS, with each sampling point placed 500 m apart in Okulu and Losekola and 1 km apart in E15 [14]. We set 41 cameras on a 4.1 km^2^ grid at Okulu between October and December 2013, 41 cameras on a 3.8 km^2^ grid at Losekola between January and November 2014, and 20 cameras on a 12.0 km^2^ grid at E15 between August 2015 and January 2016 [14]. We used Moultrie and Bushnell cameras of similar technical specifications with infrared sensors detecting change in temperature in the environment over a 24 h period.

Based on direct observations, lesula was thought to be a semi-terrestrial species, which led to the choice of positioning ground cameras on trees facing animal trails. We did not use any bait and did not select sites based on tree size or species, or the presence of fruits. The surveys were designed to capture *C. lomamiensis* behaviors; therefore, cameras were set to video mode. Date and time of capture were stamped on each video and videos were set to the highest resolution (1280 × 720 pixels).

#### 2.2.4. Data Analysis

We recorded the sampling effort as the total number of days camera traps were functional in the field. We viewed all videos (n = 1885 in Okulu, n = 9179 in Losekola, n = 3570 in E15) using MPEG Streamclip 1.9.2 (http://www.squared5.com/svideo/mpeg-streamclip-mac.html, accessed on 1 July 2016) and marked all videos containing lesula for further analysis. We trained two independent teams of undergraduate research students and volunteers to enter data. We compared lesula data from both teams to control for errors and all discrepancies (n = 27) were corrected [36].

We defined an event as a series of chronological videos with a cluster of activity from the same social group. We recorded a new event when no lesula individuals were detected within a 30 min period. We determined the capture rate by dividing the number of lesula events by the sampling effort (camera trap days) multiplied by 100 [37].

#### 2.2.5. Behavioral and Ecological Analyses

##### Degree of Terrestriality

Arboreal monkeys rarely use the forest floor for locomotion [38]. Thus, we compared the capture rates of all lesula events to those of arboreal monkey species detected by the terrestrial camera traps at the three sites. We also calculated the percentage of lesula terrestrial events, which we defined as at least one video per event, where a minimum of one animal was observed with all four limbs on the ground for at least one second.

##### Activity Pattern

By organizing all video events by time of day, we determined hourly capture rates to reveal the activity pattern of lesula over a 24 h period.

##### Birth Synchronicity

To generate estimates of infant ages and birth months, we classified all infant captures using the features listed in Table 1. Our approach followed the physical features related to age for other guenon species [39,40,41]. To increase accuracy, three observers were involved in the categorization. We discarded events for which no agreement could be made (n = 17). Precipitation data were collected using a rain gauge between 2008 and 2013 in Obenge (1°24′24.588″ S 24°59′22.128″ E), which was a former village located within the LNP boundaries (mean annual precipitation = 2021 mm; rainfall days = 145 days) (J. Hart, unpub. data). We then compared the number of births and the amount of rainfall per month.

##### Group Size and Composition

**Figure 2:**
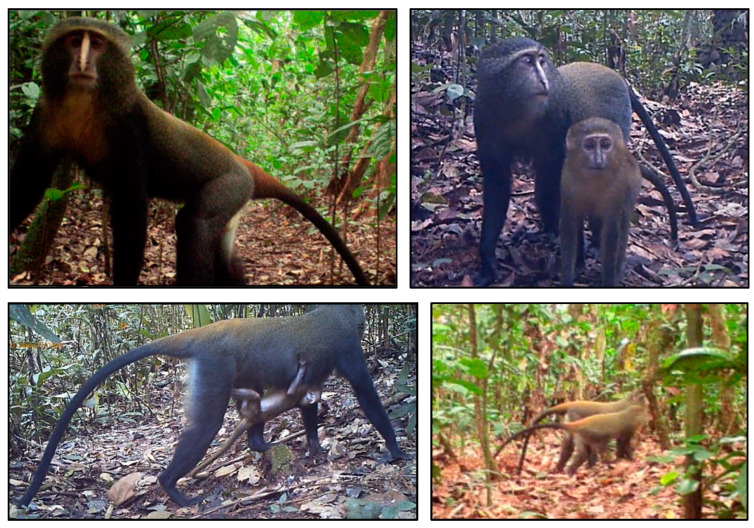
Cropped screenshots of camera trap videos of lesula (*Cercopithecus lomamiensis*) individuals in Lomami National Park and buffer zone, Democratic Republic of the Congo. (**Top left**): an adult male; (**Top right**): an adult female with juvenile; (**Bottom left**): an adult female with a clinging infant; (**Bottom right**): an adult male and adult female in close proximity showing sexual dimorphism.

We determined the group size and composition for each event by identifying a minimum number of unique individuals based on age, sex, and/or physical features, as well as their position and directionality of movement in the videos. Physical features included unique markings, such as scars and kinked tails. We estimated age-class (infant, juvenile, or subadult/adult) and sex (male, female) (Figure 2) [14]. We defined a social group as three or more individuals with at least one female or juvenile present [42]. Each group size estimate was recorded by at least two observers to ensure reliability. We combined group size data to calculate a mean group size for the three survey sites.

## 3. Results

### 3.1. Camera Trap Surveys

Data were recorded from 39, 33, and 20 cameras from Okulu, Losekola, and E15, respectively [14]. We excluded 11 cameras from the analysis due to malfunctions. Video length varied among camera traps and surveys from 20 to 90 s. We obtained a total of 598 lesula events from 5960 camera trap days over a three-year period (Table 2). Losekola had the highest capture rate of the three sites (Table 2).

### 3.2. Terrestriality

**Figure 3:**
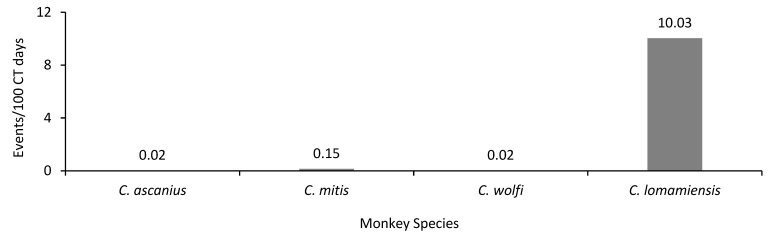
Comparison of capture rates between lesula (*C. lomamiensis*) and arboreal monkey species in LNP and buffer zone, Democratic Republic of the Congo (*C. ascanius*, n = 1 event (arboreal); *C. mitis*, n = 9 events (terrestrial); *C. wolfi*, n = 1 event (terrestrial); *C. lomamiensis*, n = 598 events (591 terrestrial events)).

Seven arboreal monkey species are known to occur at our survey sites [1]; however, our CT surveys captured only three species: *C. ascanius*, *C. mitis*, and *C. wolfi*. Lesula capture rates were 67–500 times higher than those of the arboreal monkey species (Figure 3). Lesula events met our definition of terrestriality 98.8% of the time (n = 591). In contrast, we recorded only 10 terrestrial events for the sympatric arboreal *Cercopithecus* species (*C. mitis*, n = 9; *C. wolfi*, n = 1), with one event of *C. ascanius* not meeting our definition of terrestriality.

### 3.3. Activity Pattern

**Figure 4:**
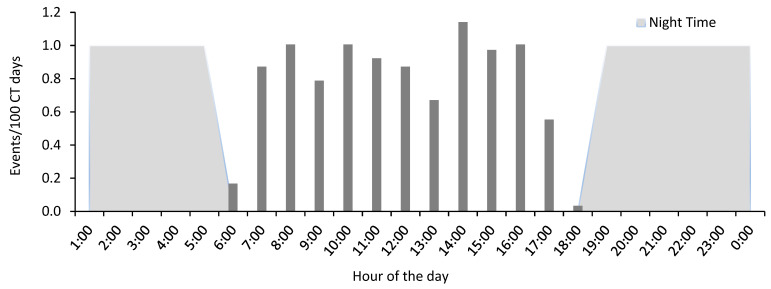
Daily activity pattern of *C. lomamiensis* showing capture rate (number of events per 100 camera trap days) calculated for each hour of the day averaged over the three surveys in Lomami National Park and buffer zone, Democratic Republic of the Congo.

All lesula events occurred between 6:00 and 19:00 h, confirming lesula’s diurnal behavior (Figure 4). Only a few events were recorded during dawn (n = 10 events between 6:00–7:00 a.m.) and dusk (n = 2 events between 6:00–7:00 p.m.). The data show a consistent pattern of activity for 11 h during the day.

### 3.4. Birth Seasonality

**Figure 5:**
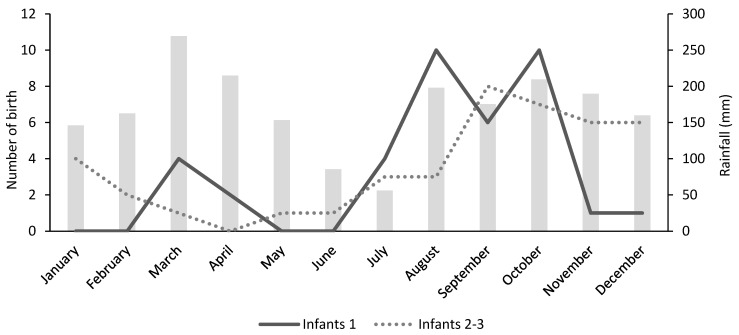
Comparison of rainfall and number of births. Average monthly rainfall in millimeters in Obenge, Democratic Republic of the Congo (S1.40683 E24.98948) inside Lomami National Park between 2008 and 2013 (J. Hart, unpub. data). Estimated number of births per month of *C. lomamiensis* averaged over the three camera trap surveys. Infant age categories are labeled regarding estimated age (1 = <1-month-old; 2 = about 3-months-old; 3 = about 6-months-old). We grouped infants 2 and 3 for analysis.

We recorded 77 events of infants with 104 independent infant sightings, representing 12.9% of all lesula events. Only infant sightings that could be assigned to the same age categories by at least two of the three observers were used to estimate the timing of the birth month, yielding 60 events with 80 infant sightings. Of the three categories used for infant classification (Table 1), we separated infant 1 records (n = 38) from infant 2 and 3 (n = 42) in the analysis. The highest peaks in births occurred from August through October, which overlaps with the start of the second wet season in the region (Figure 5).

### 3.5. Group Size

**Figure 6:**
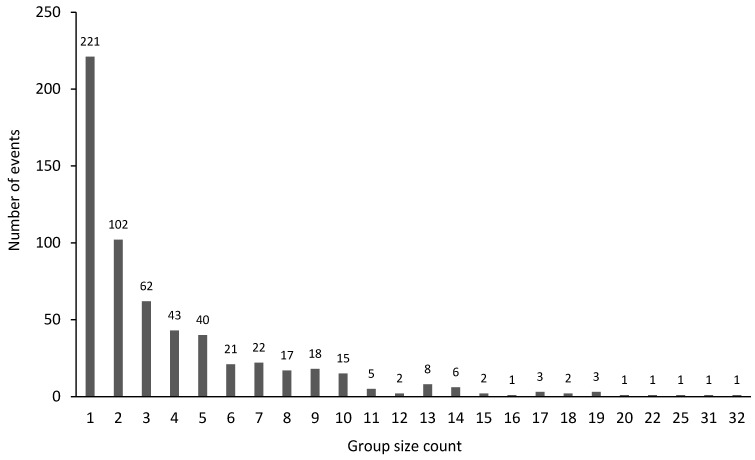
Distribution of group size counts for all lesula (*C. lomamiensis*) events (n = 598) from the three surveys combined in Lomami National Park and buffer zone, Democratic Republic of the Congo.

By considering events with group counts of three or more individuals with at least one female or juvenile identified (40% of events, n = 238), the mean group size from the three surveys combined was 7.17 individuals (±SD = 4.68). Events capturing one or two individuals represented 54% (n = 323) of all events and only 15% of the events (n = 87) had a group size larger than the mean (Figure 6). The top three largest group counts captured were 32, 31, and 25 individuals in Losekola and E15. Events were based on multiple videos 42% of the time (n = 249) with a maximum of 14 videos in an event.

### 3.6. Group Composition

Combining all surveys, we recorded a total of 2298 individuals, with a ratio of 1.67 mature individuals per immature (matures = 1163, immatures = 696), where matures comprised adult individuals (males and females), and immatures comprised juveniles and infants. We also recorded a ratio of 1.05 adult males per adult female (♂ = 147, ♀ = 140); however, we were unable to determine sex identification in most of the mature individuals (75%; n = 876). Individuals classified as unknowns, which could not be assigned to any age or sex category, represented 19% (n = 439) of all individuals captured and were excluded from ratio calculations.

Adult males were observed in 24% of events (n = 58) following our definition of a group, of which we captured nine events with two adult males in the same social group (mean group size = 14 individuals/group). Out of 188 events of one animal captures (removing individuals categorized as unknown), we recorded 49% subadults/adults of unknown sex (n = 93), 32% adult males (n = 61), 10% juveniles (n = 19), and 8% adult females (n = 15), which shows a significant difference between categories (Pearson’s chi-square = 45.32, df = 3, *p* < 0.001). Overall, 45% of all males identified were either lone males or in male-only groups (one event of two males and one event of three males) (n = 66 males).

## 4. Discussion

The high capture rates of lesula monkeys at all three survey sites indicate that camera trapping is an effective field method to detect and study the cryptic and threatened *C. lomamiensis*. The pooled event data of lesula monkeys provided new observations of this little-known guenon species, expanding our current knowledge of its behavioral ecology. We documented the following behavioral and ecological traits of lesula: terrestrial locomotion, diurnal activity pattern, birth seasonality, medium–large social groups with one–two adult males, multiple adult females and their juveniles, and likely a social organization of female philopatry and male dispersal.

Results from our behavioral study support Arenson et al. [12] findings from their morphological analysis of lesula’s post cranium that lesula is strongly terrestrial. Terrestriality in the lesula species is possibly an adaptation to competition in the higher substrates of the canopy. The forest floor of the Congo Basin may have provided the ancestors of the *hamlyni* species-group a new ecological niche to avoid competition for resources with sympatric species [43,44]. The divergence in substrate use, and foraging resources from sympatric guenon species could have promoted the evolution of the *hamlyni* species-group [45]. A productive next step for field research will be to select habitats where lesula are abundant and place camera traps at different canopy levels to assess the frequency of substrate use and the full locomotor profile of lesula [4].

Our results also indicate that lesula’s terrestrial activity pattern was exclusively confined to the daylight hours, with low activity during crepuscular hours, consistent with the diurnality of all *Cercopithecus* species [46]. Spending most of its time on the forest floor, lesula’s large orbits are potentially an adaptation to the dim forest environment [13]. Future studies incorporating arboreal surveys will be able to explore a potential link between the pre-dawn morning calls heard in the field and arboreal activity during early morning hours.

In the birth seasonality analysis, agreement between all three observers for infants 2 and 3 was challenging due to ambiguity between categories from poor quality videos. Thus, the more objective approach was to base our results on infants 1 only, where infant sightings were categorized with a high inter-observer reliability (>95%). The results suggest that the birth season of *C. lomamiensis* follows the expected pattern seen in other guenons and lasts about 4 months during the second wet season of the year [19,20,47]. This study provides preliminary results on the birth seasonality exhibited by lesula, but additional field research on reproductive behavior is needed to confirm our results. The correlation between the birth peak and rainfall is likely explained by rainfall acting as a proxy for food availability and fruiting season. Fruits are high energy food items for primates and are preferred by nursing females to successfully raise their offspring [23,48].

Our survey results suggest that lesula live in groups larger than previously reported [1] from direct observations in the field (up to 32 individuals). It is similar to closely related terrestrial species, such as *C. hamlyni*, *Allochrocebus solatus*, and *Allochrocebus lhoesti*, which live in groups of up to 22, 25, and 37 individuals, respectively [10]. However, comparison with related species suggests that the lesula mean group size (m = 7.17 ± SD 4.68) is on the low range and probably underestimated. Despite its terrestrial locomotion, individuals climb into the canopy and space themselves out when traveling, so it is difficult for ground camera traps to capture and record complete social groups. By having a limited 50-degree ground vision, the camera detection zone captures partial group counts more often than complete group counts. Indeed, events of one and two individuals composed most of the dataset and only a small part of events had a group size larger than the mean, which suggests high false detections of full social groups. We suggest using a paired camera design in future surveys to increase the detection probability of group members.

The findings reveal that lesula groups are comprised of multiple females with their infants and juveniles, which suggest a polygynous mating system. In addition, we recorded single males and male-only groups comprising up to three males, which can be identified as bachelor groups. Therefore, our results imply that lesula exhibit a female philopatry and male dispersal social organization, similar to most *Cercopithecus* species [20,24,25]. The results indicate that lesula groups mostly contain single males; however, some larger groups contained two mature males. There are four possible explanations for these observations. First, one of the males may have been in the transition of becoming sexually mature (from subadult to adult) and ready to disperse. Second, in some *Cercopithecus* species, it is common to observe multimale influxes and promiscuous mating during the breeding season [24,42,49]. One of the two males observed may have been an immigrant male that joined the group for a limited period of time (part of the mating season), when the resident male’s position was challenged by bachelor males [24]. Third, multiple males in female philopatric groups may occur when groups reach a maximum size and resident males cannot defend a high number of females. It has been noted in some *Cercopithecus* species that several sexually mature males can reside in groups for several months [50,51]. Finally, large group sizes with two adult males might represent ephemeral associations between two social groups overlapping at home range limits.

## 5. Conclusions

This study adds to our knowledge of the behavioral ecology of the lesula species, and the diversity found within the *Cercopithecus* genus. Camera trapping has been used in numerous studies to collect data on primate populations when the target species is highly cryptic and elusive [4], and/or the presence of humans can be detrimental to the animals’ wellbeing or impact their natural behaviors [52].

In forested regions where hunting occurs, there are often ethical concerns about habituating primates [53]. In these cases, camera trapping provides an alternative approach to collect observational data on threatened and understudied species, such as lesula. The remoteness of wilderness areas, such as the Lomami National Park, introduces additional challenges and costs to habituating primates. Camera trapping increases the feasibility of remote field studies as cameras can run independently when field researchers are not present. Overall, camera trap studies contribute to conservation efforts of threatened species by providing new approaches to collect life history and behavioral data, which can be applied to wildlife management.

## Figures and Tables

**Table 1:** Definitions of infant age category and estimation of birth month of *Cercopithecus lomamiensis* derived from the three camera trap surveys in Lomami National Park and buffer zone, Democratic Republic of the Congo.

Category	Definition	Birth Month Estimate
Infant 1	Newborn infant; Clinging to adult female; Skin can be seen through fine textured hair; Infant body size is relatively small compared to adult female body size (range from 1/4 to 1/3).	<1 month before event
Infant 2	Hair fully grown; Can be clinging to adult female or walking by itself where female is in proximity (both individuals captured on the same screen); If clinging, infant covers the full ventral portion of the female (about 1/2).	3 months before event
Infant 3	Independent from adult female (>1 m away); Still possesses infant physical features, behaviors, and coloring; Transitioning from infant to juvenile.	6 months before event

**Table 2:** Sampling effort (days), number of independent events, capture rate (events per 100 camera trap days) for the three different surveys in Lomami National Park and buffer zone, Democratic Republic of the Congo.

Site	Sampling Effort	Events	Capture Rate
Okulu	1551	143	9.22
Losekola	2430	282	11.61
E15	1979	173	8.74
Total	5960	598	10.03

## Data Availability

The datasets analyzed during the current study are available from the corresponding author, Kate M. Detwiler, upon reasonable request.
